# Knowledge and communication needs assessment of community health workers in a developing country: a qualitative study

**DOI:** 10.1186/1478-4491-7-59

**Published:** 2009-07-21

**Authors:** Zaeem Haq, Assad Hafeez

**Affiliations:** 1 Johns Hopkins University Centre for Communication Programs (PAIMAN), Islamabad, Pakistan; 2 Health Systems and Policy Unit, Federal Ministry of Health, Islamabad, Pakistan

## Abstract

**Background:**

Primary health care is a set of health services that can meet the needs of the developing world. Community health workers act as a bridge between health system and community in providing this care. Appropriate knowledge and communication skills of the workers are key to their confidence and elementary for the success of the system. We conducted this study to document the perceptions of these workers on their knowledge and communication needs, image building through mass media and mechanisms for continued education.

**Methods:**

Focus group discussions were held with health workers and their supervisors belonging to all the four provinces of the country and the Azad Jammu & Kashmir region. Self-response questionnaires were also used to obtain information on questions regarding their continued education.

**Results:**

About four fifths of the respondents described their communication skills as moderately sufficient and wanted improvement. Knowledge on emerging health issues was insufficient and the respondents showed willingness to participate in their continued education. Media campaigns were successful in building the image of health workers as a credible source of health information.

**Conclusion:**

A continued process should be ensured to provide opportunities to health workers to update their knowledge, sharpen communication skills and bring credibility to their persona as health educators.

## Background

Primary Health Care (PHC) defined as "Essential health care made universally accessible to individuals and families in the community by means acceptable to them, through their full participation, and at a cost that community and country can afford" has been recommended as a set of health services that can meet the challenges of a changing world [1]. The World Health Organization (WHO) in its latest report has called for a revival of PHC [2].

An important component of the rejuvenated concept of PHC is community health workers, (CHWs) who act as a bridge between the health care delivery system and the community. Mary & Rosemary have described how CHWs enable health programmes to achieve three interconnected goals: building a relationship between the health care provider and laypersons in the community; improving appropriate health care utilization; and educating people to reduce health risks in their lives [3]. Highly challenging and innovative ideas such as serving 70% of a population of 190 million in Brazil, skin-to-skin care for newborns in India and improved perinatal care in Nepal have worked remarkably well through CHWs [4-6].

Appropriate knowledge and interpersonal communication expertise, in addition to basic clinical skills, supplies and supervision, are a key to the work of CHWs [7-9]. The CHW can empower the community to identify its needs and can assist in planning a strategy to achieve the desired results. In order to accomplish this successfully, CHWs should be culturally sensitive, with an ability to build a strong community rapport.

The 100 000 Lady Health Workers (LHWs) of Pakistan's Ministry of Health fit well into the definition of CHWs; their programme is considered as one of the successful large-scale community programmes [10]. Various evaluations have enumerated the successes of this programme, along with a few areas to ensure quality improvement [11,12]. Regarding quality improvement, a number of authors recommend devising strategies to improve health worker education and training, and suggest that preferences of primary care workers should be known and discussed at the policy level [13,14].

None of the evaluations from Pakistan have sought the workers' own perceptions regarding their knowledge and communication capacity, however. We therefore conducted this qualitative study in all provinces of Pakistan, to know the perceptions of health workers and supervisors on the communication capability of the LHW; adequacy of their knowledge; effectiveness of the image-building activities involving mass media; and mechanisms for continued education.

## Methods

The Ministry of Health, Government of Pakistan, launched the National Programme for Family Planning and Primary Health Care of Pakistan, also called the Lady Health Workers Programme (LHWP) in 1994. Since then, the programme has deployed about 100 000 LHWs and more than 5000 Lady Health Supervisors (LHSs) in 135 districts of all the four provinces and regions of the country.

Providing appropriate and implementable health information to rural households has been the cornerstone of the programme's health promotion strategy. After recruitment, its workers undergo 15 months of preparation: three months of classroom training, followed by supervised fieldwork for one year. Refresher training sessions are also conducted from time to time.

There is a system of supervision meetings every month in which the 15 to 20 LHWs from the area share their progress and problems with the supervisor. The programme broadcasts issue-based communication campaigns on television and other mass media in which the LHW is positioned as an accessible and credible source of health information to the rural household.

We conducted a multi-stage, stratified, random sampling for this study. Under some of the donor-funded programmes, various initiatives for capacity building of the LHW are being carried out selectively throughout the country. To gauge the true programme situation, we selected only those districts where no donor-funded project was being implemented. These included two rural districts of Attock and Charsaddah from the provinces of Punjab and NWFP, and two urban districts of Karachi and Quetta from the provinces of Sind and Baluchistan. Muzaffarabad District was selected from the region of Azad Jammu & Kashmir (AJK), while tribal regions could not be considered because of the prevailing security situation in those areas. In each district, the sample comprised all LHWs and supervisors who were aged 20 to 50 years, based at their respective villages, married or unmarried, willing to participate in the study and having at least one year of work experience.

It was a cross-sectional study consisting of two components. Component 1 comprised focus group discussions (FGDs) with LHWs and their supervisors; in component 2, information from the same LHWs and their supervisors was obtained through a self-response questionnaire.

We developed guiding questions for the FGDs of component 1 and a self-reporting questionnaire for component 2. Careful attention was given to how the questions on "perceived barriers" would be asked during the FGD. We included appropriate examples to explain the questions uniformly across all the discussions. The questionnaires, originally developed in English, were translated to Urdu. The self-response questionnaire was back-translated as well, according to the recommendations [15]. The questionnaires were pilot-tested with groups in Rawalpindi District and appropriate changes made in the light of their feedback.

Prior appointments were made before travelling to the respective districts to conduct the discussions. Before formal discussion, the purpose of the research was explained to all the participants. The discussions held in Urdu and were tape-recorded after obtaining participants' permission. Notes were also taken, so that no discussion point was missed. Discussions were carried out with the help of guiding and probing questions. The self-reporting questionnaire was distributed among the participants at the end of each FGD. The questionnaire was first explained, after which the respondents filled in the required information and returned the questionnaires to the facilitator.

Inductive analysis [16,17] was performed on the transcripts and field notes through the following.

1. familiarization with the data, which included reading the notes, transcribing the FGDs and translating the Urdu transcript into English;

2. initial categorization by developing tables on themes emerging from the discussions. Notes taken during the discussions as well as the transcripts were consulted for developing these tables to ensure as much rigor as possible.

3. identifying patterns and connections within and between categories with the help of these tables;

4. entering data from the quantitative questionnaire into the Statistical Package for Social Sciences;

5. reaching a final interpretation by combining all the findings.

The answers of the majority were presented as the "Response", while comments that were significant but not shared by the majority were labelled as "Additional Comments". The study was undertaken between 8 March 2008 and 15 August 2008. Ethical approval for the study was obtained from the National Programme for Family Planning & Primary Health Care.

## Results

### Participants

A total of 105 participants, including 57 LHWs and 48 LHSs from five districts, participated in the research. They took part in FGDs and also filled out the questionnaire. The minimum number of participants in FGDs was seven (Karachi), while the maximum was 16 (Muzaffarabad). The mean age of the participant LHWs was 31 years (range 20–49). Among them, 88% were married, while 12% of the LHW were not married. The number of participating supervisors was 48, with a mean age of 30 years (range 23–50). Among the LHS, 92% were married, while 8% belonged to the unmarried category (Table 1).

**Table 1:** District-wise sociodemographic variables of the participants (n = 105)

	**LHW**					**LHS**				
District	No.	Age range, years	Mean age, years	Married	Unmarried	No.	Age range, years	Mean age, years	Married	Unmarried

Attock	12	24–40	30	9	3	9	27–32	30	9	0

Charsaddah	8	22–40	30	8	0	9	25–35	28	9	0

Karachi	8	28–49	34	8	0	7	23–39	32	6	1

Muzaffarabad	16	27–44	30	14	2	11	24–36	29	9	2

Quetta	13	20–45	32	11	2	12	21–50	29	11	1

Total	57	20–49	31	50	7	48	21–50	30	44	4

### Communication skills

Out of the five groups of LHWs, four believed they possessed moderately sufficient communication skills (Table 2). The group from Quetta, however, thought they possessed insufficient skills. The same proportion (four fifths) of the supervisors rated these skills as moderately sufficient, while one group (Karachi) called the communication skills of their LHW as sufficient.

**Table 2:** Responses to the questions regarding communication skills

**Question**		**LHW**	**LHS**
Are they sufficient?	Response	Moderate (4/5) Insufficient (1/5)	Sufficient (1/5) Moderate (4/5)
	
	Additional comments	There is room for improvement	LHWs with education <10 grades, not married, or those having low SES face more difficulty

How you deal with barriers perceived by individuals?	Response	By talking about child's future, using religious teachings, using help of influentials	Talking about child & family's future, using religion, using local leaders
	
	Additional comments	Using fear appeal and using help of LHS mentioned by some	Positive examples & using IEC materials, helping with own hand

What are your specific suggestions on the communications capacity building of LHW?	Response	Refresher training, role plays on common difficult scenarios, better IEC materials should be provided	Refresher training, role plays on common difficult scenarios, better IEC materials should be provided
	
	Additional comments	Adequacy and timeliness of the supply of IEC materials should be improved	Quality of basic training should also be improved

Communicating with males on family planning; establishing village health committees; convincing TB suspects to make use of diagnostic facilities; and talking about taboo subjects such as HIV/AIDS and other sexually transmitted diseases (STDs) were reported as health issues on which dialogue was difficult for the LHWs.

The respondents informed that *nazr *(evil eye), *garam & thanda *(hot & cold) food, male child preference, fear of stigma in TB and other diseases, and fatalism were the common barriers perceived by the community. Talking about the ways in which they addressed these barriers, the workers reported using better child health leading to better prospects for the family as an incentive for the people to make desired changes in their behaviour. They also reported using religious teachings where appropriate; using fear appeal; and seeking the help of influentials (teachers, counsellors, peers) where available. The main response of the majority of the supervisors was similar to that of the workers.

The respondents suggested refresher training sessions that include role plays on common difficult scenarios as a way to improve communication skills of the workers (Table 2). They proposed that appropriate information and skills to deal with people who were fixed on strong negative feelings, such as "we are poor, we can't do anything" or "a woman's only role is to serve the husband, kids and the family" or "the life or death of the mother or newborn is the will of God, in which the mortals cannot intervene" would be really helpful. The workers also suggested that information, education communication (IEC) materials should be provided to them that could be carried to the households and used for talking about specific health issues.

### Level of knowledge

Family planning (FP); maternal, newborn and child health (MNCH); nutrition; malaria; the Expanded Programme on Immunization (EPI) and common childhood diseases (Table 3) were reported as topics on which the workers had sufficient knowledge. Yet, they wanted more knowledge on some of these issues, e.g. MNCH, FP and communicable diseases such as TB. Emerging diseases such as Congo fever, avian influenza or dengue fever were reported as areas in which workers had insufficient knowledge. According to workers, their community asked questions on these emerging diseases to which they (workers) could not respond, as these topics were not a routine part of their curriculum or training.

**Table 3:** Respondents' views on adequacy of technical knowledge

**Question**	**Response**	
	
	**LHW**	**LHS**
What are the topics on which you have sufficient knowledge?	FP, MNCH, nutrition, malaria, EPI & common childhood diseases	FP, MNCH, nutrition, malaria, EPI, common childhood diseases & National Immunizations Days

What are the topics on which you have insufficient knowledge?	Emerging diseases, medicinal issues, questions on repeated weighing and polio immunization of babies are difficult topics	Emerging diseases, e.g. dengue fever, Congo fever, avian influenza, etc.

All the respondents thought some means of continuing education would help them improve their areas of weak knowledge. They liked the idea of receiving a regular publication from the programme. Out of the total, about 3% of the respondents showed interest in receiving official or administrative information and 40% were interested in reading clinical (*tibbi maalooomat*) information, while 57% wanted both types of information in equal amounts through such a publication.

**Figure 1:**
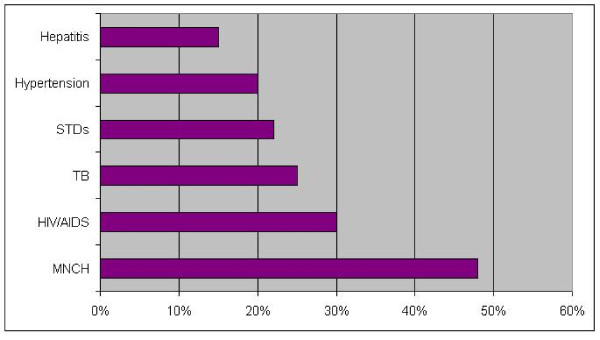
**Six priority topics on which respondents sought further information (n = 105)**.

The respondents were also asked whether a regular source of information, such as a periodical sent from the programme, would help them address the queries. A little over 94% thought such a regular source of information would help them respond to these questions (Figure 1).

### Media campaigns

Respondents belonging to both categories in all the districts liked their representation in the mass media. Table 4 describes their views as well as suggestions on these media campaigns. They thought the media enhanced the credibility of health workers as the messages on television (TV), radio or newspapers were liked by their family and community members. According to them, people believed in the information provided by the LHW when a similar message was concurrently shown on TV. The supervisors thought the community respected the worker and acknowledged her services after having seen TV commercials (TVCs) about her roles and responsibilities. The respondents suggested some modifications to improve the mass media campaigns. In their view, using the genre of television drama, adding male characters to the media products and airing these campaigns on private cable TV channels in addition to the state-run terrestrial channels would help increase the effectiveness of these campaigns.

**Table 4:** Respondents views on media campaigns and its improvement

**Question**		**LHW**	**LHS**
What are your and your family/community's views on media campaigns about the LHW programme?	Response	The campaigns please us/enhance our credibility.	The campaigns please us/enhance our credibility.
	
	Additional comments	People believe in our message when they have seen it on TV	Community relates to the worker because of these commercials

What changes should be made to improve these campaigns?	Response	Male characters should be added to TVCs. Drama format should also be tried. Multiple channels should be used	Role of LHS should be shown. Male characters should be added to TVCs.
	
	Additional comments	Community should also be shown.	Media should dispel that this programme is only about FP.

## Discussion

To our knowledge, this is the first study that has explored how community-level health workers see their own performance. The workers and their supervisors acknowledged that there was room for improvement in their communication capacity. In spite of the attention given, knowledge on some of the areas that were part of the original curriculum remained weak, while at the same time the evolving public health situation in the country demanded addition of basic information on emerging health issues to the training system. The idea of a regular publication for continuing education of these workers therefore was highly appreciated.

Dealing with barriers perceived by the community requires communication skills in addition to updated knowledge. Interestingly, without guidelines the workers were using some of the recommended techniques, e.g. use of positive examples or fear appeal [18], but they wanted more capacity to deal with these barriers. Adding role plays in the training to deal with common difficult scenarios, as suggested by the health workers and their supervisors, could help the workers.

For the LHWs, talking to male members of their community about FP topped the list of "difficult to discuss" areas; participants from all over the country reported this difficulty. Given the conservative prevailing culture and the sensitivity of the topic, this difficulty is understandable, yet talking to males – who are the sole decision-makers in the patriarchal system of society – is vitally important. Adding male mobilizers to the health education arm can be one solution. Alternatively, the recently reported techniques [19] that employed the community worker to empower a woman to discuss with her husband vital issues such as child spacing and bring about change in the FP behaviour of the couple, should also be explored.

Bringing out a regular publication for the continued education of workers and their supervisors was a novel suggestion. Owing to the low level of literacy, the LHW in Pakistan is recruited with a minimal education of eight to 10 years of schooling. A three-month classroom training session is provided, which obviously is not enough to build her capacity to remember all the details on about 20 topics on which she is expected to talk. An additional burden is imposed by emerging health issues, which are not part of her curriculum but that become popular health topics when the fear of an epidemic arises.

Given the large number of LHWs, arranging frequent refresher training sessions to help them refresh their knowledge and gain information on new health issues also has many logistical and financial implications. In the light of this research, the programme has already initiated publishing a quarterly newsletter that contains both technical and administrative information and is mailed directly to all the 100 000 workers. Such innovations can be replicated by other CHW programmes.

The mass media messages disseminated by the programme from time to time brought recognition and credibility to the worker. After watching these campaigns on mass media, the community readily believed that these workers had been hired and trained by the Ministry of Health and would bring good advice and beneficial products. Low use of CHW programmes has been linked to poor community introduction of the programme [9]. Mass media campaigns have effectively addressed this issue with regard to the CHW programme in Pakistan. These campaigns, with suggested modifications, should be continued.

This research explored the views of health workers and their supervisors qualitatively as well as quantitatively, which is the strength of this study. However, as the respondents of both components were the same, their views can be called only suggestive, and not representative of the whole population. Similarly, how the community views the knowledge and communication capacity of these workers and their perceptions about the media campaigns conducted by the programme should also be explored, to develop a better understanding of the programme, its image and the performance.

In the context of resource constraints that many health systems face today, enhancing the role of the CHW has been highlighted as an alternative strategy by various experts [20,21]. According to WHO, the key factor in shortages of professional health workers in low-income countries can be addressed by "task-shifting", which is a delegation of tasks to the "lowest" category that can perform them successfully [9]. WHO has also recommended appropriate training and adequate and continuous support for these workers in order for them to perform optimally.

According to Nigel et al. [13], every country should strive to increase the number of health workers according to its priorities, but pragmatically many low-income countries initially focused on community and mid-level workers to address the high burden of disease in the primary care setting. They describe how Thailand improved its health system through this strategy during the 1970s–1990s and countries such as Brazil, Ethiopia, Ghana, India and Malawi have adopted a similar approach.

## Conclusion

CHWs may seem elementary in high-resource settings, but they have a valuable role to play in developing countries. Some basic steps are required to facilitate them in improving their efficacy and effectiveness. A continued process should be ensured by primary health care programmes whereby opportunities are provided to community health workers to update their knowledge, sharpen communication skills and bring credibility to their persona as health educators.

## Competing interests

The authors declare that they have no competing interests.

## Authors' contributions

ZH and AH conceptualized this study; ZH carried out analysis of the data, conceptualized this paper and developed the first draft, while both authors jointly developed the final manuscript.

## References

[B1] Alma Ata Declaration. http://www.who.int/hpr/NPH/docs/declaration_almaata.pdf.

[B2] World Health Organization (2008). The World Health Report 2008: Primary Health Care Now More Than Ever Geneva.

[B3] Mary AN, Rosemary S (2003). State of evaluation: Community Health Workers. Public Health Nursing.

[B4] Bulletin of the World Health Organization. http://www.who.int/bulletin/volumes/86/4/08-030408.pdf.

[B5] Darmstadt GL, Kumar V, Yadav R, Singh V, Singh P, Mohanty S, Baqui AH, Bharti N, Gupta S, Misra RP, Awasthi S, Singh JV, Santosham M, the Saksham Study Group (2006). Introduction of community-based skin-to-skin care in rural Uttar Pradesh, India. Journal of Perinatology.

[B6] Manandhar DS, Osrin D, Shrestha BP, Mesko N, Morrison J, Tumbahangphe KM, Tamang S, Thapa S, Shrestha D, Thapa B, Shrestha JR, Wade A, Borghi J, Standing H, Manandhar M, Costello AM (2004). Effect of a participatory intervention with women's groups on birth outcomes in Nepal: cluster-randomised controlled trial. The Lancet.

[B7] Haq Z, Iqbal Z, Rahman A (2008). Job stress among community health workers: a multi-method study from Pakistan. International Journal of Mental Health Systems.

[B8] Afsar H, Younus M (2005). Recommendations to strengthen the role of lady health workers in the National Program for Family Planning and Primary Health Care in Pakistan: the health workers perspective. Journal of Ayub Medical College.

[B9] World Health Organization (2007). Community Health Workers: What Do We Know About Them?.

[B10] Haines A, Sanders D, Lehmann U, Rowe AK, Lawn JE, Jan S, Walker DG, Bhutta Z (2007). Achieving child survival goals: potential contribution of community health workers. The Lancet.

[B11] Ministry of Health Government of Pakistan (2008). Internal Assessment of Lady Health Workers' Programme 2007.

[B12] Oxford Policy Management (2002). External Eevaluation of the National Programme for Family Planning and Primary Health Care: Summary of Final Report Oxford.

[B13] Crisp N, Gawanas B, Imogen S (2008). Training the health workforce: scaling up, saving lives. The Lancet.

[B14] Manongi RN, Marchant TC, Bygbjerg IC (2006). Improving motivation among primary health care workers in Tanzania: a health worker perspective. Human Resources for Health.

[B15] Rahman A, Iqbal Z, Waheed W, Husain N (2003). Translation and cultural adaptation of health questionnaires. Journal of Pakistan Medical Association.

[B16] Pope C, Zieblan S, Mays N (2000). Qualitative research in health care: analyzing qualitative data. BMJ.

[B17] Taylor-Powell E, Renner M Analyzing qualitative data. University of Wisconsin-Extension Program Development & Evaluation. http://www.uwex.edu/ces/pdande.

[B18] Witte K (2000). A meta analysis of fear appeals: implications for effective public health campaigns. Health Education & Behavior.

[B19] Rahman A, Malik A, Sikander S, Roberts C, Creed F (2008). Cognitive behaviour therapy-based intervention by community health workers for mothers with depression and their infants in rural Pakistan: a cluster-randomised controlled trial. The Lancet.

[B20] Macinko J, de Souza MF, Guanais FC, Simoes CC (2007). Going to scale with community-based primary care: an analysis of the family health program and infant mortality in Brazil, 1994–2004. Social Sciences & Medicine.

[B21] World Health Organization (2006). The World Health Report 2006: Working Together For Health Geneva.

